# Full-Genome Analysis of Influenza A(H7N9) Virus from Shanghai, China, 2014

**DOI:** 10.1128/genomeA.00578-14

**Published:** 2014-06-19

**Authors:** Wanju Zhang, Yanchao He, Lei Xu, Fahui Dai, Zhoufang Mei, Ling Qian, Desheng Xie, Ying Shen, Yong Gu, Zhiyong Zhang, Zhenghong Yuan, Zhijun Jie, Yunwen Hu

**Affiliations:** a Pathogen Diagnosis and Biosafety Department, Shanghai Public Health Clinical Center, Fudan University, Shanghai, China; b Department of Respiratory Medicine, Fifth People’s Hospital of Shanghai, Fudan University, Shanghai, China; c Key Laboratory of Medical Molecular Virology, School of Basic Medical Science, Shanghai Medical College of Fudan University, Shanghai, China

## Abstract

We analyzed the complete genome sequence of the A/Shanghai/01/2014 (H7N9) strain, which will provide a better understanding of the evolution of influenza A(H7N9) virus.

## GENOME ANNOUNCEMENT

Influenza A virus (IAV), which is a member of the genus *Influenzavirus*, family *Orthomyxoviridae*, contains 8 single-stranded negative-sense RNA (-ssRNA) segments that encode 12 proteins. Recently, a novel avian-origin influenza A(H7N9) virus has caused >410 cases of infection in China, including 60 deaths from March 2013 to 8 April 2014 (1). Influenza A(H7N9) cases worldwide were first found in the Minhang district of Shanghai in 2013 (2). No more cases were discovered in this area after May 2013, until the first Shanghai H7N9 case of 2014 reemerged in the same area in January. The patient, an 86-year-old man, was admitted to the Fifth People’s Hospital of Shanghai with a 4-day history of fever (up to 40°C) with cough on 30 December 2013. The throat swabs and sputum samples were collected on the day of admission and day 4 after admission. Viral RNA extracted from the samples was subjected to real-time reverse transcription-PCR (RT-PCR) for detecting influenza type A and subtype A(H7N9) according to the protocol provided by the Chinese CDC (3, 4). Viral RNA extracted from the supernatant of the sputa was subjected to amplify each of the viral gene segments using the One-Step RT-PCR kit (Qiagen, Inc.) using a set of gene-specific primers. The PCR products were directly sequenced with an ABI 3730XL automatic DNA analyzer using the ABI Prism BigDye Terminator cycle sequencing kit 3.1.

All gene segments of the A/Shanghai/01/2014 (H7N9) virus were compared with those of the vaccine component strain A/Anhui1/1/2013 and those of the currently circulating strains. The complete coding region of A/Shanghai/01/2014 (H7N9) is 13,090 nucleotides long. Segments 1 to 8 are 2,280, 2,274, 2,151, 1,683, 1,497, 1,398, 969, and 838 nucleotides (nt), respectively. The strain has an amantadine resistance mutation (S31N substitution) in the viral M2 protein and no oseltamivir resistance mutation (R292K substitution) in the viral neuraminidase (NA) protein (5). The mutation encoding E627K in the polymerase basic 2 (PB2) protein, which confers high virulence to avian influenza in mammalian hosts (6), was not found.

The similarities of the PB1, polymerase acidic (PA), hemagglutinin (HA), nucleoprotein (NP), NA, matrix (M), and nonstructural (NS) genes between the sequence of A/Shanghai/01/2014 (H7N9) and the consensus sequence of 2013 strains (*n* = 34) were all >99.9%, while the similarity of the PB2 gene was only 96.8%. Phylogenetic analysis indicated that the PB2 gene of the A/Shanghai/01/2014 (H7N9) virus was not clustered with those of the human H7N9 strains in 2013 but clustered with those of A/Chicken/Wenzhou/598/2013 (H9N2) and A/Chicken/Wenzhou/642/2013 (H9N2). The similarities of the PB2 nucleic acid sequences between the A/Shanghai/01/2014 (H7N9) and A/Chicken/Wenzhou/598/2013 (H9N2) strains were as high as 99.6%, which suggests that the origin of the PB2 gene fragment in the A/Shanghai/01/2014 (H7N9) virus strain was different from those of human strains in 2013. The sequence information reported here will facilitate further investigations of the evolution of human infected avian-origin H7N9 influenza virus.

### Nucleotide sequence accession numbers.

The complete genome sequence of the A/Shanghai/01/2014 (H7N9) strain has been deposited and updated in GenBank under the accession no. KJ411975 to KJ411982.
